# Neuroinvasive *Bacillus cereus* Infection in Immunocompromised Hosts: Epidemiologic Investigation of 5 Patients With Acute Myeloid Leukemia

**DOI:** 10.1093/ofid/ofae048

**Published:** 2024-01-25

**Authors:** Jessica S Little, Cassie Coughlin, Candace Hsieh, Meaghan Lanza, Wan Yi Huang, Aishwarya Kumar, Tanvi Dandawate, Robert Tucker, Paige Gable, Axel A Vazquez Deida, Heather Moulton-Meissner, Valerie Stevens, Gillian McAllister, Thomas Ewing, Maria Diaz, Janet Glowicz, Marisa L Winkler, Nicole Pecora, David W Kubiak, Jeffrey C Pearson, Marlise R Luskin, Amy C Sherman, Ann E Woolley, Christina Brandeburg, Barbara Bolstorff, Eileen McHale, Esther Fortes, Matthew Doucette, Sandra Smole, Craig Bunnell, Anne Gross, Dana Platt, Sonali Desai, Karen Fiumara, Nicolas C Issa, Lindsey R Baden, Chanu Rhee, Michael Klompas, Meghan A Baker

**Affiliations:** Harvard Medical School, Boston, Massachusetts, USA; Dana-Farber Cancer Institute, Boston, Massachusetts, USA; Division of Infectious Diseases, Brigham and Women's Hospital, Boston, Massachusetts, USA; Harvard Medical School, Boston, Massachusetts, USA; Dana-Farber Cancer Institute, Boston, Massachusetts, USA; Department of Infection Control, Brigham and Women's Hospital, Boston, Massachusetts, USA; Harvard Medical School, Boston, Massachusetts, USA; Dana-Farber Cancer Institute, Boston, Massachusetts, USA; Department of Infection Control, Brigham and Women's Hospital, Boston, Massachusetts, USA; Harvard Medical School, Boston, Massachusetts, USA; Dana-Farber Cancer Institute, Boston, Massachusetts, USA; Department of Infection Control, Brigham and Women's Hospital, Boston, Massachusetts, USA; Harvard Medical School, Boston, Massachusetts, USA; Dana-Farber Cancer Institute, Boston, Massachusetts, USA; Department of Infection Control, Brigham and Women's Hospital, Boston, Massachusetts, USA; Harvard Medical School, Boston, Massachusetts, USA; Dana-Farber Cancer Institute, Boston, Massachusetts, USA; Department of Infection Control, Brigham and Women's Hospital, Boston, Massachusetts, USA; Dana-Farber Cancer Institute, Boston, Massachusetts, USA; Department of Infection Control, Brigham and Women's Hospital, Boston, Massachusetts, USA; Harvard Medical School, Boston, Massachusetts, USA; Department of Infection Control, Brigham and Women's Hospital, Boston, Massachusetts, USA; Division of Healthcare Quality Promotion, Centers for Disease Control and Prevention, Atlanta, Georgia, USA; Division of Healthcare Quality Promotion, Centers for Disease Control and Prevention, Atlanta, Georgia, USA; Epidemic Intelligence Service, Centers for Disease Control and Prevention, Atlanta, Georgia, USA; Division of Healthcare Quality Promotion, Centers for Disease Control and Prevention, Atlanta, Georgia, USA; Division of Healthcare Quality Promotion, Centers for Disease Control and Prevention, Atlanta, Georgia, USA; Division of Healthcare Quality Promotion, Centers for Disease Control and Prevention, Atlanta, Georgia, USA; Division of Healthcare Quality Promotion, Centers for Disease Control and Prevention, Atlanta, Georgia, USA; Division of Healthcare Quality Promotion, Centers for Disease Control and Prevention, Atlanta, Georgia, USA; Division of Healthcare Quality Promotion, Centers for Disease Control and Prevention, Atlanta, Georgia, USA; Harvard Medical School, Boston, Massachusetts, USA; Division of Infectious Diseases, Brigham and Women's Hospital, Boston, Massachusetts, USA; Division of Microbiology, Brigham and Women's Hospital, Boston, Massachusetts, USA; Harvard Medical School, Boston, Massachusetts, USA; Division of Microbiology, Brigham and Women's Hospital, Boston, Massachusetts, USA; Division of Infectious Diseases, Brigham and Women's Hospital, Boston, Massachusetts, USA; Department of Pharmacy, Brigham and Women's Hospital, Boston, Massachusetts, USA; Division of Infectious Diseases, Brigham and Women's Hospital, Boston, Massachusetts, USA; Department of Pharmacy, Brigham and Women's Hospital, Boston, Massachusetts, USA; Harvard Medical School, Boston, Massachusetts, USA; Dana-Farber Cancer Institute, Boston, Massachusetts, USA; Harvard Medical School, Boston, Massachusetts, USA; Dana-Farber Cancer Institute, Boston, Massachusetts, USA; Division of Infectious Diseases, Brigham and Women's Hospital, Boston, Massachusetts, USA; Harvard Medical School, Boston, Massachusetts, USA; Dana-Farber Cancer Institute, Boston, Massachusetts, USA; Division of Infectious Diseases, Brigham and Women's Hospital, Boston, Massachusetts, USA; Massachusetts Department of Public Health, Boston, Massachusetts, USA; Massachusetts Department of Public Health, Boston, Massachusetts, USA; Massachusetts Department of Public Health, Boston, Massachusetts, USA; Massachusetts Department of Public Health, Boston, Massachusetts, USA; Massachusetts Department of Public Health, Boston, Massachusetts, USA; Massachusetts Department of Public Health, Boston, Massachusetts, USA; Harvard Medical School, Boston, Massachusetts, USA; Dana-Farber Cancer Institute, Boston, Massachusetts, USA; Harvard Medical School, Boston, Massachusetts, USA; Dana-Farber Cancer Institute, Boston, Massachusetts, USA; Harvard Medical School, Boston, Massachusetts, USA; Dana-Farber Cancer Institute, Boston, Massachusetts, USA; Harvard Medical School, Boston, Massachusetts, USA; Department of Quality and Safety, Brigham and Women's Hospital, Boston, Massachusetts, USA; Harvard Medical School, Boston, Massachusetts, USA; Department of Infection Control, Brigham and Women's Hospital, Boston, Massachusetts, USA; Harvard Medical School, Boston, Massachusetts, USA; Dana-Farber Cancer Institute, Boston, Massachusetts, USA; Division of Infectious Diseases, Brigham and Women's Hospital, Boston, Massachusetts, USA; Harvard Medical School, Boston, Massachusetts, USA; Dana-Farber Cancer Institute, Boston, Massachusetts, USA; Division of Infectious Diseases, Brigham and Women's Hospital, Boston, Massachusetts, USA; Division of Infectious Diseases, Brigham and Women's Hospital, Boston, Massachusetts, USA; Department of Infection Control, Brigham and Women's Hospital, Boston, Massachusetts, USA; Department of Population Medicine, Harvard Medical School, Harvard Pilgrim Healthcare Institute, Boston, Massachusetts, USA; Division of Infectious Diseases, Brigham and Women's Hospital, Boston, Massachusetts, USA; Department of Infection Control, Brigham and Women's Hospital, Boston, Massachusetts, USA; Department of Population Medicine, Harvard Medical School, Harvard Pilgrim Healthcare Institute, Boston, Massachusetts, USA; Dana-Farber Cancer Institute, Boston, Massachusetts, USA; Division of Infectious Diseases, Brigham and Women's Hospital, Boston, Massachusetts, USA; Department of Infection Control, Brigham and Women's Hospital, Boston, Massachusetts, USA; Department of Population Medicine, Harvard Medical School, Harvard Pilgrim Healthcare Institute, Boston, Massachusetts, USA

**Keywords:** acute myeloid leukemia, *Bacillus cereus*, immunocompromised, infection control investigation, neuroinvasive infection

## Abstract

**Background:**

*Bacillus cereus* is a ubiquitous gram-positive rod-shaped bacterium that can cause sepsis and neuroinvasive disease in patients with acute leukemia or neutropenia.

**Methods:**

A single-center retrospective review was conducted to evaluate patients with acute leukemia, positive blood or cerebrospinal fluid test results for *B cereus*, and abnormal neuroradiographic findings between January 2018 and October 2022. Infection control practices were observed, environmental samples obtained, a dietary case-control study completed, and whole genome sequencing performed on environmental and clinical *Bacillus* isolates.

**Results:**

Five patients with *B cereus* neuroinvasive disease were identified. All patients had acute myeloid leukemia (AML), were receiving induction chemotherapy, and were neutropenic. Neurologic involvement included subarachnoid or intraparenchymal hemorrhage or brain abscess. All patients were treated with ciprofloxacin and survived with limited or no neurologic sequelae. *B cereus* was identified in 7 of 61 environmental samples and 1 of 19 dietary protein samples—these were unrelated to clinical isolates via sequencing. No point source was identified. Ciprofloxacin was added to the empiric antimicrobial regimen for patients with AML and prolonged or recurrent neutropenic fevers; no new cases were identified in the ensuing year.

**Conclusions:**

*B cereus* is ubiquitous in the hospital environment, at times leading to clusters with unrelated isolates. Fastidious infection control practices addressing a range of possible exposures are warranted, but their efficacy is unknown and they may not be sufficient to prevent all infections. Thus, including *B cereus* coverage in empiric regimens for patients with AML and persistent neutropenic fever may limit the morbidity of this pathogen.

## Teaser

We describe 5 health care–associated neuroinvasive *Bacillus cereus* infections. No point source was identified, and isolates were unrelated. Enhancing environmental cleaning, laundry management, and the neutropenic diet, as well as modifying empiric treatment protocols for febrile neutropenia, aborted the cluster.

## INTRODUCTION

*Bacillus cereus* is a ubiquitous gram-positive or gram-variable spore-forming rod-shaped bacterium that causes serious invasive infections in immunocompromised hosts [1–9]. In patients with acute leukemia and neutropenia, *B cereus* infection can lead to sepsis and neuroinvasive disease with a range of neurologic complications, such as meningoencephalitis, cerebritis, brain abscess, cerebral infarction, cerebral hemorrhage, and subarachnoid hemorrhage. Neuroinvasive *B cereus* infections are frequently fatal despite prompt administration of appropriate antimicrobial therapy [1, 10, 11]. Typical β-lactam therapy, such as cefepime or piperacillin-tazobactam administered for febrile neutropenia, will not treat *B cereus* due to its β-lactamase production. Recommended therapies are vancomycin, carbapenems, or fluoroquinolones, although isolates resistant to each of these antimicrobial classes have been reported [12–16]. *B cereus* is abundant in the environment and is a common contaminant in microbiologic culture [1, 8, 17]. However, it is critical not to overlook its potential clinical significance in high-risk populations. It has been associated with nosocomial outbreaks related to hospital construction, contaminated linens and towels, faulty ventilation systems, parenteral nutrition formulas, and dietary exposures such as tea and probiotics [1, 2, 18–22]. Clusters of genetically unrelated cases co-occurring in time without a point source have also been described.

A previous cluster of neuroinvasive *B cereus* infections was identified at our institution in 2013 to 2014 prompting an epidemiologic investigation. No clear point source was identified, as all isolates were unrelated; however, there was evidence linking cases to *Bacillus* contamination of unpeeled bananas associated with nearby construction [1]. Subsequent mitigating measures were instituted, such as adjustment of the neutropenic diet, enhanced environmental cleaning practices, and a temporary change to the fever and neutropenia protocol, which included the addition of empiric ciprofloxacin therapy for patients with acute myeloid leukemia (AML) receiving induction chemotherapy with persistent or recurrent neutropenic fever. Following the discontinuation of these measures, 3 patients with AML developed health care–associated neuroinvasive *B cereus* infections in close temporal proximity in 2022, triggering an extensive epidemiologic investigation that identified 2 additional cases between 2018 and 2022.

## METHODS

**Figure 1.**
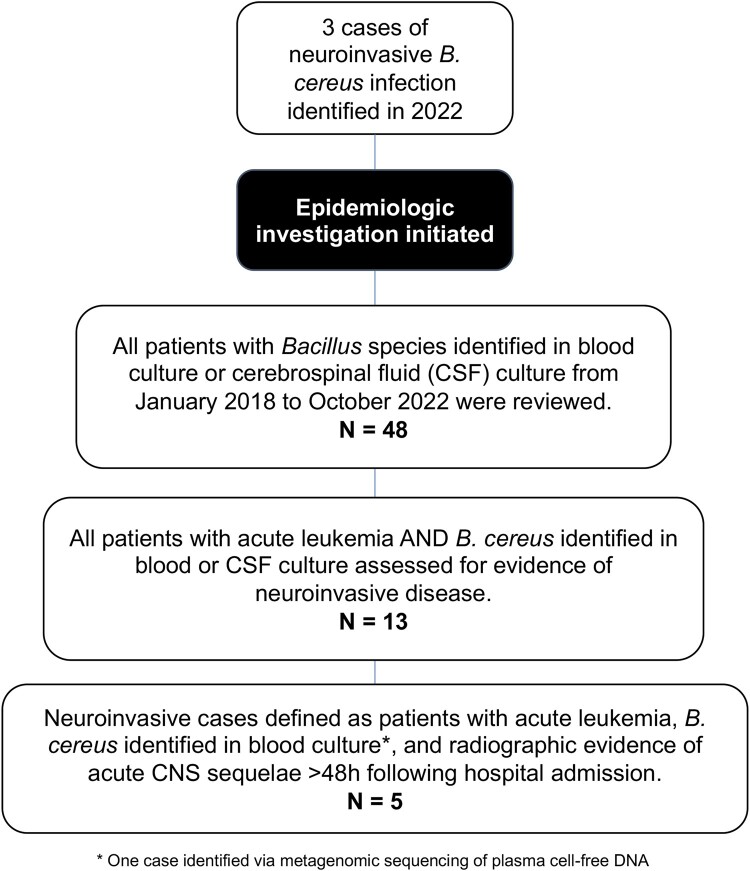
Identification of neuroinvasive *Bacillus cereus* infections. The figure describes the methods for identifying neuroinvasive *B cereus* infections. Cases were defined as patients with a diagnosis of acute leukemia, positive microbiologic cultures (blood or cerebrospinal fluid) for *B cereus* ≥48 hours after admission, and correlating central nervous system radiographic findings. After 3 index cases were identified, all patients with microbiologic cultures positive for *Bacillus* species were assessed (n = 48). Cases of patients with a diagnosis of acute leukemia and a positive culture with *B cereus* during the risk period were extensively reviewed (n = 13), including 5 that exhibited correlating abnormal central nervous system radiographic findings consistent with neuroinvasive disease.

We identified all patients with acute leukemia and health care–associated neuroinvasive *B cereus* infection at Brigham and Women's Hospital and Dana-Farber Cancer Institute between January 2018 and October 2022 (Figure 1). After 3 index cases were identified, all patients with *B cereus* isolated from blood or cerebrospinal fluid (CSF) via microbiologic culture or molecular testing ≥48 hours after admission were assessed (n = 48). Standard microbiologic methods were used to identify *Bacillus* species on blood culture. Patients with a diagnosis of acute leukemia and *B cereus* isolated via microbiologic testing during the risk period were extensively reviewed (n = 13), including 5 that exhibited correlating abnormal central nervous system (CNS) radiographic findings. Given the high barrier to pursue lumbar puncture or brain biopsy in this population with profound neutropenia and thrombocytopenia, case definitions consisted of those with positive blood cultures, suggestive clinical history, and new abnormal CNS radiographic findings within 2 weeks of diagnosis, even in the absence of positive CSF cultures or confirmatory CNS histopathology. Patients were followed through last follow-up as of 1 August 2023. Clinical information was collected from the electronic medical record: demographics, medical history, presenting symptoms, microbiologic and radiographic studies, laboratory testing, treatment, and outcomes. Infection control practices were evaluated on wards where patients with hematologic malignancy received care. Environmental samples were obtained from patient rooms (exhaust ducts), portable medical equipment (laundry cart, blanket warmer), and linens (towels, sheets, pillowcases, blankets, and unused patient gowns at multiple points during the complete laundry process from the commercial laundry through the delivery to patient care floors). Sixty-one samples were collected from surfaces via environmental swabs or RODAC agar plates (replicate organism detection and counting; blood agar, 5%, contact plates with Lok-Tight friction lids; Hardy Diagnostics) with a contact time of 10 seconds per plate [23, 24]. Hospital construction projects during the at-risk period were assessed, and patient locations were noted. A matched case-control study was performed to assess dietary risk factors for *B cereus* infection. Fourteen control patients without *Bacillus* infections were randomly selected among patients with AML admitted for induction chemotherapy between October 2021 and August 2022. Odds ratios and *P* values were calculated with a Fisher exact test. Whole genome sequencing (WGS) was performed on the available clinical *Bacillus* isolates (n = 2), 15 environmental *Bacillus* isolates recovered from 7 environmental samples, and 2 isolates recovered from a sample of dietary protein supplement common to most patients with AML. Sequencing was performed by Brigham and Women's Hospital, the Centers for Disease Control and Prevention, and the Massachusetts Department of Public Health [25], respectively. FASTQ files were shared with the Massachusetts Department of Public Health for bioinformatic analysis, which was performed as previously described with modifications outlined in the supplementary methods [26, 27]. Institutional review board approval was not sought because this study was done as part of a public health and infection control investigation.

## RESULTS

### Clinical Characteristics of Neuroinvasive *B cereus* Infection

Five patients with AML and *B cereus* neuroinvasive disease were identified between January 2018 and November 2022. Clinical characteristics of cases are presented in Table 1. The median age was 41 years (range, 24–55) and 60% were male. All patients were hospitalized for intensive cytotoxic induction chemotherapy (cytarabine and daunorubicin with venetoclax, n = 3; cytarabine and daunorubicin with crenolanib, n = 1; FLAG-IDA [fludarabine, cytarabine, granulocyte-stimulating colony factor, and idarubicin], n = 1) and were neutropenic at the time of infection. Four cases were identified via blood culture, and 1 case (patient 3) was identified via metagenomic sequencing of plasma cell-free DNA (Karius) [28]. No cases were identified via CSF culture or CNS tissue histopathology. Susceptibility testing (E-test method) on 4 available isolates showed a minimum inhibitory concentration of 4 μg/mL for vancomycin and <0.25 μg/mL for ciprofloxacin for all isolates.

**Table 1.** Clinical Characteristics of Neuroinvasive *Bacillus cereus* Infections

						Symptoms		Radiographic Findings		Treatment	
No.	Age, y	Sex	Induction Regimen	No. of Positive Cultures	Antimicrobial MICs	Presenting	Neurologic	CNS	Other	Initial Combined Regimen^a^	Ciprofloxacin Duration, mo
1	≥50	M	Cytarabine + daunorubicin + venetoclax	2 sets (4/4 bottles)	Vancomycin, 4; ciprofloxacin, 0.25	Fever, diarrhea, abnormal liver function test results	Encephalopathy, right CN VII palsy, right hemiparesis	Left basal ganglia cerebral hemorrhage	Hepatic microabscesses	Vancomycin, ciprofloxacin	8
2	<50	M	FLAG-IDA	2 sets (4/4 bottles)	Vancomycin, 4; ciprofloxacin, 0.06	Diarrhea, hypotension	None	Rim-enhancing right internal capsule lesion	Enteritis	Vancomycin, ciprofloxacin	2
3	<50	F	Cytarabine + daunorubicin + crenolanib	mNGS (Karius)	NR	Fever, nausea, abdominal pain	Headache, photophobia, meningismus	Left frontal lobe rim-enhancing lesion with hemorrhagic focus	None	Vancomycin, meropenem	8
4	<50	F	Cytarabine + daunorubicin + venetoclax	4 sets (7/8 bottles)	Vancomycin, 4; ciprofloxacin, 0.25	Fever, diarrhea, abdominal pain	Headache	Right and left frontal subarachnoid hemorrhage	Typhlitis, hepatic microabscesses	Vancomycin, ciprofloxacin	2
5	≥50	M	Cytarabine + daunorubicin + venetoclax	2 sets (4/4 bottles)	Vancomycin, 4; ciprofloxacin, 0.06	Fever, diarrhea, rectal pain	Encephalopathy, left hemiparesis, aphasia	Right and left frontal cerebral hemorrhage	Typhlitis, colitis, hypodense hepatic lesions	Vancomycin,meropenem, ciprofloxacin	5

Each patient had acute myeloid leukemia, and each 6-month outcome was survival.

Abbreviations: CN, cranial nerve; CNS, central nervous system; F, female; FLAG-IDA, fludarabine, cytarabine, granulocyte-stimulating colony factory, and idarubicin; M, male; MIC, minimum inhibitory concentration; mNGS, metagenomic next-generation sequencing; NR, not reported.

^a^The final treatment regimen was ciprofloxacin for all patients.

**Figure 2.**
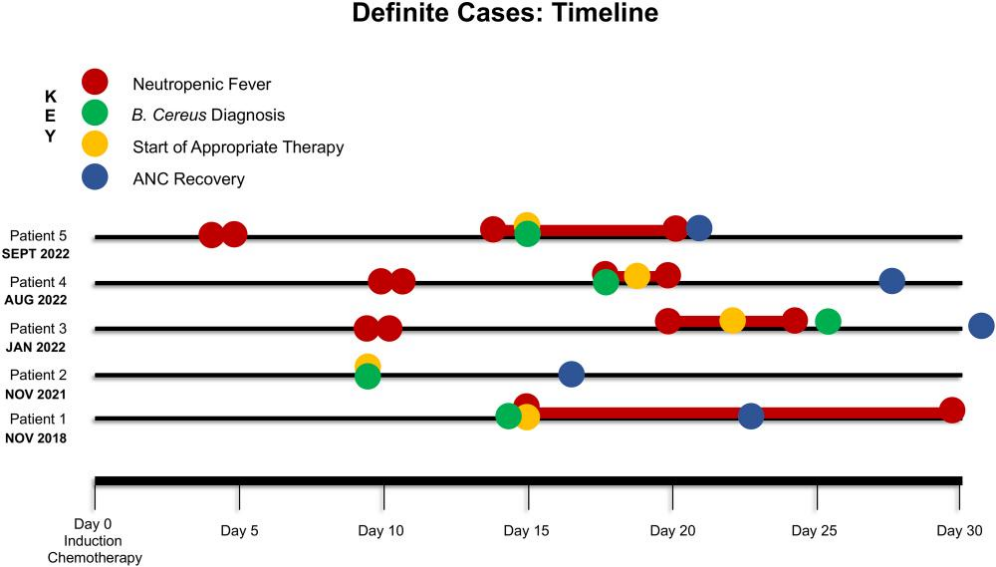
Timeline of neuroinvasive *Bacillus cereus* infections. The figure demonstrates the timing of neuroinvasive *B cereus* infections, including first and recurrent fever, diagnosis of *B cereus* bacteremia, and recovery of neutrophils after intensive cytotoxic induction chemotherapy. ANC, absolute neutrophil count.

*B cereus* infection occurred most often 1 to 3 weeks after initiation of chemotherapy, with a median time to diagnosis of 14 days from the first day of chemotherapy (range, 8–26 days; Figure 2). Fever was the presenting symptom in 4 of 5 patients (80%), and all patients had gastrointestinal symptoms that preceded their neurologic complications, such as diarrhea (n = 4) and/or abdominal/rectal pain (n = 3). Neurologic symptoms were headache (n = 2), encephalopathy (n = 2), focal neurologic deficits (cranial nerve palsy, n = 1; hemiparesis, n = 2; aphasia, n = 1), and meningismus (n = 1). CNS sequelae occurred at a median of 1 day (range, 0–11) after positive microbiologic diagnosis and were highly morbid: 60% (n = 3) developed intraparenchymal or subarachnoid hemorrhage and 40% (n = 2) had rim-enhancing lesions or brain abscesses. Three patients (60%) also had radiographic evidence of enteritis or typhlitis, and 3 (60%) had evidence of hepatic hypodense lesions or “microabscesses” on computed tomography imaging of the abdomen, although these were not microbiologically confirmed to be related to *B cereus* infection. All patients had prior exposure to β-lactam antibiotics, including cefepime (n = 5) and piperacillin-tazobactam (n = 2). Notably, 2 patients had received fluoroquinolone prophylaxis before infection was detected; however, their fluoroquinolones were discontinued at the time of first neutropenic fever (8 and 13 days prior to diagnosis), and they transitioned to an anti-*Pseudomonal* β-lactam antibiotic, per the hospital febrile neutropenia guidelines.

Following diagnosis, all patients were treated with combination therapy for 1 to 4 weeks with ciprofloxacin, vancomycin, or meropenem. Patients ultimately transitioned to ciprofloxacin monotherapy at hospital discharge and completed a median 147 days (range, 54–220) of total therapy. Three patients (60%) had radiographic worsening of CNS lesions following recovery of neutrophils, one of whom (patient 3) underwent brain biopsy to exonerate a secondary or new process that demonstrated negative microbiologic studies. Patient 5 received a time-limited course of corticosteroids for presumed immune reconstitution inflammatory syndrome. All patients received additional cycles of chemotherapy without clinical worsening, and 80% successfully underwent hematopoietic cell transplantation at a median 109 days (range, 100–204) after *B cereus* neuroinvasive infection. All patients recovered without gross residual neurologic deficits, though one patient had mild persistent neurocognitive deficits [1].

### Epidemiologic Investigation of Neuroinvasive *B cereus* Infections

**Figure 3.**
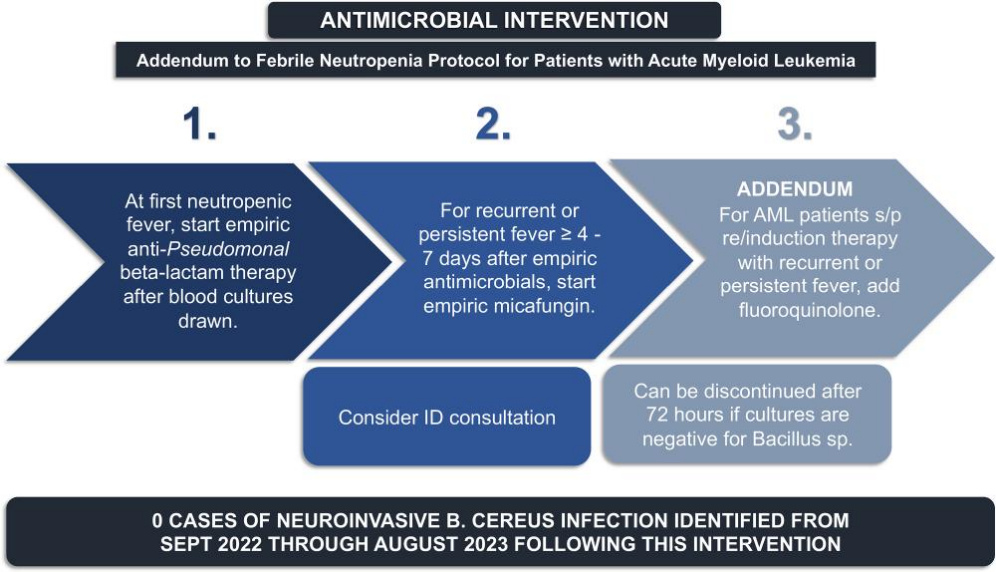
Empiric antimicrobial strategy for prevention of neuroinvasive *Bacillus cereus* infections in patients with acute myeloid leukemia. The figure displays the empiric antimicrobial strategy that was initiated at our institution among patients with acute myeloid leukemia for the prevention of neuroinvasive *B cereus* infection. The strategy was elected to maximize antimicrobial stewardship by ensuring time-limited courses of empiric fluoroquinolone therapy and prompt discontinuation if blood cultures remained negative and there was no evidence of neuroinvasive *B cereus* infection. Fluoroquinolone therapy was selected given optimal central nervous system penetration and borderline elevated minimum inhibitory concentrations to vancomycin for the organisms isolated at our institution. AML, acute myeloid leukemia.

The 5 patients were on 3 separate oncology wards, housed on one floor of the hospital at the time of infection. There was no overlap in patient rooms. From the targeted environmental sampling, *B cereus* was identified in 7 of 61 environmental samples, including 2 laundry cart covers, a laundry cart shelf, an exhaust duct in a patient bathroom, and unused linens (flat sheet, fitted sheet, blanket) stored on a laundry cart on the oncology ward. *B cereus* was not isolated from any of the linen samples collected from the laundry facility. Renovation and construction projects at the hospital were assessed with no correlation to the location or timing of the cases. On case-control analysis of 5 patients with neuroinvasive *B cereus* and 14 controls, no dietary exposures were significantly associated with *B cereus* infection. All patients received a dietary protein supplement, which was previously identified as a potential source of *B cereus* contamination: samples were therefore obtained from 19 lots [1]. *B cereus* was identified in 1 of 19 samples that were tested; however, the concentration of bacterial growth fell under the US Food and Drug Administration thresholds for dietary supplements. WGS analysis of the dietary protein isolate and environmental specimens did not show any relationship to available clinical *B cereus* isolates (n = 2). Two environmental isolates were highly related via WGS (bathroom exhaust duct and unused fitted sheet). Empiric fluoroquinolone therapy was added back to the antimicrobial regimen for patients with AML receiving induction or reinduction chemotherapy with prolonged or recurrent neutropenic fevers in September 2022 for prevention of neuroinvasive *B cereus* infections (Figure 3). No new cases of neuroinvasive infection have since been identified through August 2023.

## DISCUSSION

Neuroinvasive *B cereus* infections are life-threatening events that affect immunocompromised hosts and can lead to death or long-term disability if not treated promptly. Here we report 5 cases of neuroinvasive *B cereus* infection that occurred in patients with AML at our institution between 2018 and 2022, with 3 in close temporal proximity in 2022. While this study was limited by the lack of microbiologic confirmation of *B cereus* in the CNS, the clinical history and temporal proximity of CNS complications to *B cereus* bloodstream infections (median, 1 day from diagnosis) was highly suggestive of neuroinvasive disease. No point source was identified, and WGS did not identify a relationship between clinical and environmental isolates. This is suggestive of sporadic infections from varying exposures in a high-risk population. Notably, while the cluster reported by Rhee et al had a high mortality rate (80%), all patients in this cluster survived with almost complete resolution of neurologic deficits and went on to receive additional immunosuppressive chemotherapy and/or hematopoietic cell transplantation [1]. This may have been related to increased familiarity with this clinical syndrome and prompt administration of appropriate antimicrobials associated with the prior cases in 2013 to 2014. A permanent empiric antimicrobial strategy was adopted after identification of these new cases in September 2022, with a fluoroquinolone added for any persistent or recurrent neutropenic fever in patients with AML who were receiving induction or reinduction chemotherapy and with no further cases reported since then (through January 2024).

*B cereus* is ubiquitous in the environment and has been associated with outbreaks in health care settings related to hospital construction, contaminated medical equipment, linens, and dietary exposures [2, 13, 18–21]. In the prior case series of 5 patients from our institution, there was no point source identified, as infections were caused by multiple distinct strains. Cranberry juice and bananas were identified as dietary exposures associated with increased risk of *B cereus* neuroinvasive infection in a case-control study. *B cereus* was also cultured from the shelf where bananas were stored and from a banana peel, raising concern that this could have represented a potential source, though these isolates were unrelated to the clinical isolates. Other environmental samples that tested positive in that study included a blanket warmer, an air sample near a construction site, and a sample of a protein dietary supplement [1]. In the current investigation, there were no associated dietary exposures, patient exposures to hospital construction areas, or a link between clinical and environmental/dietary *B cereus* isolates on WGS. In this case-control study, all patients received a protein dietary supplement for nutritional purposes, and 19 lots were tested for *B cereus*, given the concern in 2015, with only 1 lot returning positive with a concentration that fell within the Food and Drug Administration’s threshold for dietary supplements. The occurrence of multiple cases without an epidemiologic link likely reflects the ubiquity of *Bacillus* spp in the environment and the potential for vulnerable patients to be exposed via multiple routes. However, this analysis was limited to the available clinical isolates (n = 2), and we cannot rule out that an epidemiologic link could exist with environmental sources that were not sampled or clinical isolates that were not included. In addition, the dietary case-control study consisted only of patients with AML with neuroinvasive *B cereus* infection instead of all of those with *B cereus* bacteremia, which may have provided additional information on dietary exposures. Despite these limitations, the ubiquity of *Bacillus* spp highlights the importance of optimizing infection control practices across multiple domains to minimize the risk of nosocomial *Bacillus* spp infections (construction management, fastidious handling of linens, protecting food from contamination and/or decreasing bioburden through irradiation, and rigorous environmental cleaning). Furthermore, it may be impossible to eliminate *Bacillus* spp entirely from the hospital environment, underlining the importance of clinical countermeasures in addition to infection control, including early recognition of the syndrome of invasive *B cereus* infections and early use of fluoroquinolones or other agents active against *Bacillus* species.

Shifts in epidemiology have led to an increasing prevalence of gram-positive bloodstream infections, including *B cereus*, in patients with hematologic malignancy, and neuroinvasive *B cereus* infections are well described in patients with acute leukemia [4, 7, 29]. Early identification and treatment of these infections are key to improving patient outcomes, though confirming a diagnosis of neuroinvasive disease can be challenging in patients with profound neutropenia and thrombocytopenia. In fact, the clinical association of neurologic events with invasive *B cereus* infection has primarily been described through postmortem studies [4, 5, 8, 30, 31]. For patients who survive, *B cereus* is often identified only on blood culture, as was the case in this series [3, 15]. Thus, a high index of clinical suspicion must be maintained for neurologic complications in patients who are immunocompromised with *B cereus* bloodstream infections. Distinct clinical features of *B cereus* neuroinvasive infections may aid in identifying the clinical syndrome even without confirmatory CSF or CNS histopathology. All patients with neuroinvasive infections at our institution had a diagnosis of AML and were receiving intensive cytotoxic induction chemotherapy, with profound neutropenia and disruption of the gastrointestinal mucosa that likely predisposed to this infection. There were no cases among patients with acute lymphoid leukemia, which could be related to lower numbers of these patients at our institution or to differences in the depth and duration of neutropenia and mucosal disruption related to chemotherapy in this population. In terms of the clinical syndrome, fever typically occurs >1 week after initiation of chemotherapy, most often as a recurrent or persistent fever despite initiation of anti-*Pseudomonal* β-lactam therapy for febrile neutropenia [1, 5, 7, 30]. Gastrointestinal symptoms, such as diarrhea, abdominal pain, and vomiting, are frequently present prior to onset of neurologic symptoms, and in our cluster, 3 patients had radiographic evidence of typhlitis/enteritis. Others have reported gastrointestinal necrosis and ulceration with *B cereus* on immunohistochemistry on autopsy [1, 31–33]. Abdominal symptoms may reflect a dietary source of inoculation with subsequent translocation leading to systemic infection. Liver involvement can also occur, with hepatic microabscesses seen on imaging in 3 patients in this series (though not microbiologically confirmed to be due to *B cereus*) and with hepatic necrosis with invasive gram-positive bacteria reported on pathologic specimens in prior published cases [1, 4, 32, 34]. These early clinical findings may raise suspicion for *B cereus* infection and can prompt additional workup.

For patients with a suggestive clinical syndrome or a known *B cereus* bloodstream infection, rapid initiation of empiric or targeted antimicrobials may be lifesaving. *B cereus* produces β-lactamases, rendering them resistant to many antibiotics typically included in empiric regimens for neutropenic fever [12, 14, 35]. Vancomycin, fluoroquinolones, and carbapenems are the empiric agents of choice for patients with suspected *B cereus* infection, and they are often used in combination for severe cases until susceptibility results return, as resistance has been reported to each class [13, 15, 34, 36, 37]. Among our cases, all patients received combination therapy initially, and no resistance to these agents was documented. However, given the relatively elevated minimum inhibitory concentration of 4 to vancomycin with the potential for reduced susceptibility, fluoroquinolones were chosen as the empiric regimen for treatment of persistent or recurrent febrile neutropenia in patients with AML who were receiving chemotherapy. Our empiric antimicrobial strategy is described in Figure 3 and was elected with several key aims. First, appropriate antimicrobial therapy must be rapidly initiated at onset of symptoms in patients at risk for *B cereus* infections in hopes of preventing the progression to neuroinvasive disease. This was balanced by a distinct goal to maximize antimicrobial stewardship and prevent widespread long-term use of fluoroquinolones, with the potential for generation of antimicrobial resistance as well as complications such as *Clostridioides difficile* colitis. This was attained by targeting only the most high-risk patients with AML who were undergoing intensive cytotoxic chemotherapy, as well as recommending a time-limited duration of antimicrobials with prompt discontinuation once *B cereus* infection was exonerated as a cause of fevers [38, 39].

The neurologic complications of *B cereus* infection are diverse: brain abscess, meningoencephalitis, intraparenchymal or subarachnoid hemorrhage, and cerebral infarct [8, 9, 11]. While the mechanism of the CNS complications is not well elucidated, it has been postulated that they are related to the production of tissue-destructive toxins or exoenzymes [35, 40]. Regardless, these events are morbid and often difficult to identify as sequelae of *B cereus* infection. In addition, patients with neurologic sequelae should be monitored closely at the time of neutrophil recovery, as clinical worsening with increased edema associated with immune reconstitution has been described and did occur in 60% of the patients in our study, with 1 patient undergoing an invasive biopsy to rule out other potential causes [7, 15, 16]. The role of corticosteroids in this setting are unknown, although patient 5 received a corticosteroid taper with clinical improvement and near full resolution of neurologic deficits.

Clinicians should have a high index of suspicion for neuroinvasive *B cereus* infections in neutropenic cases with hematologic malignancy that include persistent or recurrent fevers in conjunction with gastrointestinal symptoms, followed by the development of new neurologic symptoms or CNS radiographic abnormalities. These infections may be underrecognized, and even though they have been associated with nosocomial outbreaks, due to the ubiquity of *B cereus*, they can also occur as sporadic infections associated with independent environmental or dietary exposures. Regardless, the morbidity and mortality of this syndrome are high; thus, infection control and clinical mitigation measures may be required. Use of additional antimicrobials, such as fluoroquinolones, to empirically treat *B cereus* infection in the right host could reduce morbidity, but these measures should be targeted to the highest-risk populations to balance the increasing risks of antimicrobial resistance and the need for stewardship in patients with hematologic malignancy.

## Supplementary Data

Supplementary materials are available at *Open Forum Infectious Diseases* online. Consisting of data provided by the authors to benefit the reader, the posted materials are not copyedited and are the sole responsibility of the authors, so questions or comments should be addressed to the corresponding author.

## Supplementary Material

ofae048_Supplementary_Data

## Notes

***Author contributions*.** J. S. L. aided in the epidemiologic investigation, assisted in drafting the empiric antimicrobial strategy, collected data on case patients, and wrote the manuscript. C. C., C. H., M. L., W. Y. H., A. K., T. D., R. T., P. G., A. A. V. D., H. M.-M., V. S., G. M., T. E., M. Diaz, J. G., M. L. W., N. P., D. W. K., J. C. P., M. R. L., A. C. S., A. E. W., N. C. I., L. R. B., C. Brandeburg, B. B., E. M., E. F., M. Doucette, S. S., C. Bunnell, A. G., D. P., S. D., K. F., C. R., and M. K. assisted in the epidemiologic investigation and edited the manuscript. L. R. B., N. C. I., A. E. W., and A. C. S. assisted in drafting the empiric antimicrobial strategy. P. G., H. M.-M., V. S., G. M., T. E., M. Diaz, C. Brandeburg , B. B., E. M., E. F., and M. Doucette assisted in performing the WGS and bioinformatics analysis. M. A. B. led the epidemiologic investigation, assisted in drafting the empiric antimicrobial strategy, collected data on case patients, and edited the manuscript.

***Data availability.*** The data that support the findings of this study are available from the corresponding author, J. S. L., upon reasonable request.

***Patient consent statement.*** This study did not include factors requiring patient consent. The study design was carried out in line with ethical standards. Institutional review board approval was not sought because this study was done as part of a public health and infection control investigation.

***Financial support*.** No funding sources.

## References

[1] Rhee C, Klompas M, Tamburini FB, et al Epidemiologic investigation of a cluster of neuroinvasive *Bacillus cereus* infections in 5 patients with acute myelogenous leukemia. Open Forum Infect Dis 2015; 2:ofv096. 26269794 10.1093/ofid/ofv096 PMC4531223

[2] Campbell JR, Hulten K, Baker CJ. Cluster of *Bacillus* species bacteremia cases in neonates during a hospital construction project. Infect Control Hosp Epidemiol 2011; 32:1035–8. 21931256 10.1086/661910

[3] Tusgul S, Prod’hom G, Senn L, Meuli R, Bochud PY, Giulieri SG. *Bacillus cereus* bacteraemia: comparison between haematologic and nonhaematologic patients. New Microbes New Infect 2017; 15:65–71. 28050250 10.1016/j.nmni.2016.11.011 PMC5192042

[4] Brouland JP, Sala N, Tusgul S, Rebecchini C, Kovari E. *Bacillus cereus* bacteremia with central nervous system involvement: a neuropathological study. Clin Neuropathol 2018; 37:22–7. 29035192 10.5414/NP301041

[5] Strittmatter M, Hamann G, Sahin U, Feiden W, Kohl K, Schimrigk K. Multiple brain abscesses and intracerebral hemorrhage caused by *Bacillus cereus* in a case of acute lymphatic leukemia. Eur J Neurol 1996; 3:149–52. 7501096

[6] Cotton DJ, Gill VJ, Marshall DJ, Gress J, Thaler M, Pizzo PA. Clinical features and therapeutic interventions in 17 cases of *Bacillus* bacteremia in an immunosuppressed patient population. J Clin Microbiol 1987; 25:672–4. 3571476 10.1128/jcm.25.4.672-674.1987 PMC266057

[7] Schoenfeld D, Lee D, Arrington JA, Greene J, Klinkova O. *Bacillus cereus* bacteremia complicated by brain abscess in a severely immunocompromised patient: addressing importance of early recognition and challenges in diagnosis. IDCases 2022; 29:e01525. 35712054 10.1016/j.idcr.2022.e01525 PMC9194585

[8] Vodopivec I, Rinehart EM, Griffin GK, et al A cluster of CNS infections due to *B cereus* in the setting of acute myeloid leukemia: neuropathology in 5 patients. J Neuropathol Exp Neurol 2015; 74:1000–11. 26352989 10.1097/NEN.0000000000000244

[9] Gaur AH, Patrick CC, McCullers JA, et al *Bacillus cereus* bacteremia and meningitis in immunocompromised children. Clin Infect Dis 2001; 32:1456–62. 11317247 10.1086/320154

[10] Ginsburg AS, Salazar LG, True LD, Disis ML. Fatal *Bacillus cereus* sepsis following resolving neutropenic enterocolitis during the treatment of acute leukemia. Am J Hematol 2003; 72:204–8. 12605393 10.1002/ajh.10272

[11] Herrera MRM, González-Urdiales P, Zubizarreta-Zamalloa A, Rodríguez-Merino E, Martínez-Dubarbie F. Central nervous system infection by *Bacillus cereus*: a case report and literature review. Rev Neurol 2022; 75:239–45. 36218254 10.33588/rn.7508.2021412 PMC10280720

[12] Luna VA, King DS, Gulledge J, Cannons AC, Amuso PT, Cattani J. Susceptibility of *Bacillus anthracis*, *Bacillus cereus*, *Bacillus mycoides*, *Bacillus pseudomycoides* and *Bacillus thuringiensis* to 24 antimicrobials using Sensititre automated microbroth dilution and Etest agar gradient diffusion methods. J Antimicrob Chemother 2007; 60:555–67. 17586563 10.1093/jac/dkm213

[13] Kalpoe JS, Hogenbirk K, van Maarseveen NM, et al Dissemination of *Bacillus cereus* in a paediatric intensive care unit traced to insufficient disinfection of reusable ventilator air-flow sensors. J Hosp Infect 2008; 68:341–7. 18358564 10.1016/j.jhin.2008.01.017

[14] Turnbull PCB, Sirianni NM, LeBron CI, et al MICs of selected antibiotics for *Bacillus anthracis*, *Bacillus cereus*, *Bacillus thuringiensis*, and *Bacillus mycoides* from a range of clinical and environmental sources as determined by the Etest. J Clin Microbiol 2004; 42:3626–34. 15297508 10.1128/JCM.42.8.3626-3634.2004 PMC497625

[15] Vidanaral AH, Kasper K, Karlowsky J, Walkty A. Successful treatment of *Bacillus cereus* group brain abscesses with a combination of high-dose ciprofloxacin and vancomycin. J Assoc Med Microbiol Infect Dis Canada 2017; 2:64–8.

[16] Koizumi Y, Okuno T, Minamiguchi H, Hodohara K, Mikamo H, Andoh A. Survival of a case of *Bacillus cereus* meningitis with brain abscess presenting as immune reconstitution syndrome after febrile neutropenia—a case report and literature review. BMC Infect Dis 2020; 20:15. 31906936 10.1186/s12879-019-4753-1 PMC6945728

[17] Drobniewski FA . *Bacillus cereus* and related species. Clin Microbiol Rev 1993; 6:324–38. 8269390 10.1128/cmr.6.4.324 PMC358292

[18] Ohsaki Y, Koyano S, Tachibana M, et al Undetected *Bacillus* pseudo-outbreak after renovation work in a teaching hospital. J Infect 2007; 54:617–22. 17145080 10.1016/j.jinf.2006.10.049

[19] Hosein IK, Hoffman PN, Ellam S, et al Summertime *Bacillus cereus* colonization of hospital newborns traced to contaminated, laundered linen. J Hosp Infect 2013; 85:149–54. 23927924 10.1016/j.jhin.2013.06.001

[20] Kniehl E, Becker A, Forster DH. Pseudo-outbreak of toxigenic *Bacillus cereus* isolated from stools of three patients with diarrhoea after oral administration of a probiotic medication. J Hosp Infect 2003; 55:33–8. 14505607 10.1016/s0195-6701(03)00133-6

[21] Cheng VCC, Chen JHK, Leung SSM, et al Seasonal outbreak of *Bacillus* bacteremia associated with contaminated linen in Hong Kong. Clin Infect Dis 2017; 64:S91–7. 28475782 10.1093/cid/cix044

[22] El Saleeby CM, Howard SC, Hayden RT, McCullers JA. Association between tea ingestion and invasive *Bacillus cereus* infection among children with cancer. Clin Infect Dis 2004; 39:1536–9. 15546093 10.1086/425358

[23] Jones CL . Guidelines for the assessment of viable fungal hygiene on indoor surfaces using RODAC Petri plates. J Bacteriol Mycol Open Access 2019; 7:116‒26.

[24] Eagle Analytical. Proper environmental surface sampling with agar plates. Available at: https://eagleanalytical.com/surface-sampling-agar-plates/. Accessed 28 September 2023.

[25] Centers for Disease Control and Prevention. Laboratory standard operating procedure for whole genome sequencing on MISEQ. Available at: https://www.aphl.org/programs/food_safety/Documents/PNL38_WGS%20on%20MiSeq%20SOP_v3.pdf. Accessed 10 June 2023.

[26] Oakeson KF, Wagner JM, Mendenhall M, Rohrwasser A, Atkinson-Dunn R. Bioinformatic analyses of whole-genome sequence data in a public health laboratory. Emerg Infect Dis 2017; 23:1441–5. 28820135 10.3201/eid2309.170416 PMC5572866

[27] Oakeson KF, Wagner JM, Rohrwasser A, Atkinson-Dunn R. Whole-genome sequencing and bioinformatic analysis of isolates from foodborne illness outbreaks of *Campylobacter jejuni* and *Salmonella enterica*. J Clin Microbiol 2018; 56:161–79. 10.1128/JCM.00161-18 PMC6204689 30158193

[28] Blauwkamp TA, Thair S, Rosen MJ, et al Analytical and clinical validation of a microbial cell-free DNA sequencing test for infectious disease. Nat Microbiol 2019; 4:663–74. 30742071 10.1038/s41564-018-0349-6

[29] Zinner SH . Changing epidemiology of infections in patients with neutropenia and cancer: emphasis on gram-positive and resistant bacteria. Clin Infect Dis 1999; 29:490–4. 10530434 10.1086/598620

[30] Marley EF, Saini NK, Venkatraman C, Orenstein JM. Fatal *Bacillus cereus* meningoencephalitis in an adult with acute myelogenous leukemia. South Med J 1995; 88:969–72. 7660218 10.1097/00007611-199509000-00017

[31] Arnaout MK, Tamburro RF, Bodner SM, et al *Bacillus cereus* causing fulminant sepsis and hemolysis in two patients with acute leukemia. J Pediatr Hematol Oncol 1999; 21:431–5. 10524460 10.1097/00043426-199909000-00018

[32] Akiyama N, Mitani K, Tanaka Y, et al Fulminant septicemic syndrome of *Bacillus cereus* in a leukemic patient. Intern Med 1997; 36:221–6. 9144019 10.2169/internalmedicine.36.221

[33] Le Scanff J, Mohammedi I, Thiebaut A, Martin O, Argaud L, Robert D. Necrotizing gastritis due to *Bacillus cereus* in an immunocompromised patient. Infection 2006; 34:98–9. 16703301 10.1007/s15010-006-5019-6

[34] Kiyomizu K, Yagi T, Yoshida H, et al Fulminant septicemia of *Bacillus cereus* resistant to carbapenem in a patient with biphenotypic acute leukemia. J Infect Chemother 2008; 14:361–7. 18936889 10.1007/s10156-008-0627-y

[35] Bottone EJ . *Bacillus cereus*, a volatile human pathogen. Clin Microbiol Rev 2010; 23:382–98. 20375358 10.1128/CMR.00073-09 PMC2863360

[36] Sakai C, Iuchi T, Ishii A, Kumagai K, Takagi T. *Bacillus cereus* brain abscesses occurring in a severely neutropenic patient: successful treatment with antimicrobial agents, granulocyte colony-stimulating factor and surgical drainage. Intern Med 2001; 40:654–7. 11506311 10.2169/internalmedicine.40.654

[37] Kobayashi K, Kami M, Ikeda M, et al Fulminant septicemia caused by *Bacillus cereus* following reduced-intensity umbilical cord blood transplantation. Haematologica 2005; 90:e13–4. 15653460

[38] Verlinden A, Jansens H, Goossens H, et al Clinical and microbiological impact of discontinuation of fluoroquinolone prophylaxis in patients with prolonged profound neutropenia. Eur J Haematol 2014; 93:302–8. 24750350 10.1111/ejh.12345

[39] Rangaraj G, Granwehr BP, Jiang Y, Hachem R, Raad I. Perils of quinolone exposure in cancer patients: breakthrough bacteremia with multidrug-resistant organisms. Cancer 2010; 116:967–73. 20052728 10.1002/cncr.24812

[40] Turnbull PC, Jørgensen K, Kramer JM, Gilbert RJ, Parry JM. Severe clinical conditions associated with *Bacillus cereus* and the apparent involvement of exotoxins. J Clin Pathol 1979; 32:289–93. 107202 10.1136/jcp.32.3.289 PMC1145637

