# Supplementary material for: Neuroinvasive Bacillus cereus Infection in Immunocompromised Hosts: Epidemiologic Investigation of 5 Patients With Acute Myeloid Leukemia
Source: Open Forum Infect Dis. 2024 Jan 25;11(3):ofae048. doi: 10.1093/ofid/ofae048 (PMC10906701; doi:10.1093/ofid/ofae048)
Supplement: ofae048_Supplementary_Data [file ofae048_supplementary_data.docx]

**Neuroinvasive *Bacillus cereus* Infection Amongst Immunocompromised Patients: Epidemiologic Investigation of 5 Cases in Patients with Acute Myeloid Leukemia**

Jessica S. Little^1,2,3^, Cassie Coughlin^1,2,4^, Candace Hsieh^1,2,4^, Meaghan Lanza^1,2,4^, Wan Yi Huang^1,2,4^, Aishwarya Kumar^1,2,4^, Tanvi Dandawate^2,4^, Robert Tucker^1,4^, Paige Gable^5^, Axel A. Vazquez Deida^5,6^, Heather Moulton-Meissner^5^, Valerie Stevens^5^, Gillian McAllister^5^, Thomas Ewing^5^, Maria Diaz^5^, Janet Glowicz^5^, Marisa L. Winkler^1,3,7^, Nicole Pecora,^1,7^ David W. Kubiak^3,8^, Jeffrey C. Pearson^3,8^, Marlise R. Luskin^1,2,^, Amy C. Sherman^1,2,3^, Ann E. Woolley^1,2,3^, Christina Brandeburg^9^, Barbara Bolstorff^9^, Eileen McHale^9^, Esther Fortes^9^, Matthew Doucette^9^, Sandra Smole^9^, Craig Bunnell^1,2^, Anne Gross^1,2^, Dana Platt^1,2^, Sonali Desai^1,4^, Karen Fiumara^1,4^, Nicolas C. Issa^1,2,3^, Lindsey R. Baden^1,2,3^, Chanu Rhee^1,2,3,4^, Michael Klompas^1,2,3,4^, Meghan A. Baker^1,2,3,4^

^1^ Harvard Medical School, Boston, MA

^2^ Dana-Farber Cancer Institute, Boston, MA

^3^ Division of Infectious Diseases, Brigham and Women's Hospital, Boston, MA

^4^ Department of Infection Control, Brigham and Women's Hospital, Boston, MA

^5^ Division of Healthcare Quality Promotion, Centers for Disease Control and Prevention, Atlanta, GA

^6^ Epidemic Intelligence Service, Centers for Disease Control and Prevention, Atlanta, GA

^7^ Division of Microbiology, Brigham and Women's Hospital, Boston, MA

^8^ Department of Pharmacy, Brigham and Women’s Hospital, Boston, MA

^9^ Massachusetts Department of Public Health, Boston, MA

**Short title:** Neuroinvasive *Bacillus cereus* Infection Amongst Immunocompromised Patients

**Word count:** 2995 (3000 max) **Abstract:** 246 (250 max)

**Tables:** 1 **Figures:** 3 **References:** 40 max

**Short summary:** (40 word max) We describe five healthcare-associated neuroinvasive *Bacillus cereus* infections. No point source was identified, and isolates were unrelated. Enhancing environmental cleaning, laundry management, enhancement of the neutropenic diet as well as modifying empiric treatment protocols for febrile neutropenia aborted the cluster.

***Correspondence to:**

Jessica S. Little, M.D.

Brigham and Women’s Hospital

75 Francis Street, PBB-A4

Boston, MA 02115

Email: [jlittle@bwh.harvard.edu](mailto:jlittle@bwh.harvard.edu)

**Supplementary Methods:**

Bioinformatics Analysis was performed by Massachusetts Department of Public Health as previously described.[1,2] Raw paired-end read quality control and adapter removal was performed using SeqyClean v1.10.09.[3] Read processing and quality evaluation was performed using CG-pipeline.[4] Organism Prediction was done using Mash v2.1.[5] Genome assembly and assembly quality evaluation performed using Shovill v1.0.4 (SPAdes v3.12.0) and QUAST 5.0.2.[6,7] Genome annotation was done using Prokka v1.14.0.[8] Pan genome generation, core genome alignment and phylogenetic analysis performed using Roary v3.12.0 and IQ-TREE v1.6.12.[9,10] High-quality single nucleotide polymorphism (hqSNP) analysis was performed using lyve-SET v1.1.4f.[11]

**References:**

1. Oakeson KF, Wagner JM, Rohrwasser A, Atkinson-Dunn R. Whole-genome sequencing and bioinformatic analysis of isolates from foodborne illness outbreaks of campylobacter jejuni and salmonella enterica. J Clin Microbiol **2018**; 56:161–179. Available at: https://journals.asm.org/doi/10.1128/jcm.00161-18. Accessed 9 October 2023.

2. Oakeson KF, Wagner JM, Mendenhall M, Rohrwasser A, Atkinson-Dunn R. Bioinformatic analyses of whole-genome sequence data in a public health laboratory. Emerg Infect Dis **2017**; 23:1441–1445. Available at: https://wwwnc.cdc.gov/eid/article/23/9/17-0416_article. Accessed 9 October 2023.

3. Zhbannikov IY, Hunter SS, Foster JA, Settles ML. Seqyclean: A pipeline for high-throughput sequence data preprocessing. In: ACM-BCB 2017 - Proceedings of the 8th ACM International Conference on Bioinformatics, Computational Biology, and Health Informatics. New York, NY, USA: ACM, 2017: 407–416. Available at: http://github.com/ibest/seqyclean. Accessed 10 October 2023.

4. Kislyuk AO, Katz LS, Agrawal S, et al. A computational genomics pipeline for prokaryotic sequencing projects. Bioinformatics **2010**; 26:1819–1826. Available at: https://dx.doi.org/10.1093/bioinformatics/btq284. Accessed 10 October 2023.

5. Ondov BD, Treangen TJ, Melsted P, et al. Mash: Fast genome and metagenome distance estimation using MinHash. Genome Biol **2016**; 17:1–14. Available at: https://genomebiology.biomedcentral.com/articles/10.1186/s13059-016-0997-x. Accessed 10 October 2023.

6. Bankevich A, Nurk S, Antipov D, et al. SPAdes: A new genome assembly algorithm and its applications to single-cell sequencing. Journal of Computational Biology **2012**; 19:455–477. Available at: https://pubmed.ncbi.nlm.nih.gov/22506599/. Accessed 10 October 2023.

7. Gurevich A, Saveliev V, Vyahhi N, Tesler G. QUAST: quality assessment tool for genome assemblies. Bioinformatics **2013**; 29:1072–1075. Available at: https://dx.doi.org/10.1093/bioinformatics/btt086. Accessed 10 October 2023.

8. Seemann T. Prokka: Rapid prokaryotic genome annotation. Bioinformatics **2014**; 30:2068–2069. Available at: https://dx.doi.org/10.1093/bioinformatics/btu153. Accessed 10 October 2023.

9. Nguyen LT, Schmidt HA, Von Haeseler A, Minh BQ. IQ-TREE: A fast and effective stochastic algorithm for estimating maximum-likelihood phylogenies. Mol Biol Evol **2015**; 32:268–274. Available at: https://dx.doi.org/10.1093/molbev/msu300. Accessed 10 October 2023.

10. Page AJ, Cummins CA, Hunt M, et al. Roary: Rapid large-scale prokaryote pan genome analysis. Bioinformatics **2015**; 31:3691–3693. Available at: https://dx.doi.org/10.1093/bioinformatics/btv421. Accessed 10 October 2023.

11. Katz LS, Griswold T, Williams-Newkirk AJ, et al. A comparative analysis of the Lyve-SET phylogenomics pipeline for genomic epidemiology of foodborne pathogens. Front Microbiol **2017**; 8:248940.
